# Campaign-style governance of air pollution in China? A comprehensive analysis of the central environmental protection inspection

**DOI:** 10.3389/fpubh.2023.1081573

**Published:** 2023-03-21

**Authors:** Yanchao Feng, Chuan Cheng, Shilei Hu, Anqi Cao

**Affiliations:** ^1^Business School, Zhengzhou University, Zhengzhou, China; ^2^School of Economics and Management, Harbin Institute of Technology, Weihai, China

**Keywords:** central environmental protection inspection, air pollution, campaign-style governance, regression discontinuity design, government-business relations

## Abstract

Central environmental protection inspection (CEPI) is a major institutional innovation in China's environmental governance, but its effectiveness in improving air quality is still unclear. However, the effectiveness of CEPI is of great significance and can be regarded as an important reference for deepening the reform of environmental governance system in China. This article takes the CEPI as a quasi-natural experiment and uses the regression discontinuity design (RDD) and the difference-in-differences (DID) methods to examine the effectiveness of this policy. The study found that the first round of CEPI reduced the air pollution of cities in the inspected provinces in a short time. Moreover, this positive policy effect persisted in the aftermath of the inspection, but this long-term effect is mainly reflected in PM_10_ and SO_2._ Heterogeneity analysis showed that CEPI was only effective in reducing air pollutants of industry-oriented cities, cities in Central and Eastern China, and cities with large or small population size. The moderating effect analysis indicated that a healthy relationship (close and clean) between the local governments and businesses was conducive to reducing air pollution. The research confirmed the presence of “selective” reduction of air pollutants in the long run caused by CEPI, thereby providing new inspiration for the improvement of campaign-style environmental governance and the follow-up CEPI work.

## 1. Introduction

Since the reform and opening-up in 1978, China has experienced vigorous development both in economic and social aspects. However, the extensive economic growth caused severe environmental deterioration. According to the *China Ecological Environment Bulletin* (CEEB) in 2021, 43.1% of China's 339 cities exceeded the national air quality standards. Moreover, among the severe and above pollution days, the number of days with PM_2.5_ as the primary pollutant accounted for 39.7%, while the number of days with PM_10_ as the main pollutant accounted for 25.2%. Evidently, the current environmental pollution in China is quite serious. Moreover, there are some deficiencies in China's current environmental protection system. To solve the problem of environmental pollution and mitigate obstacles rooted in China's environmental governance, the Chinese government formulated and implemented a series of environmental protection policies, regulations, and measures, such as environmental protection inspection and environmental protection interview. Among them, central environmental protection inspection (CEPI) emerged as the times required during the 13th Five Year Plan (FYP) period. Compared with the traditional environmental monitoring system, CEPI has wider coverage, higher inspection level and stricter punishment.

Once the CEPI policy was promulgated, it attracted the attention of the academic community. Scholars have mostly studied the reasons for the success of CEPI, the problems and the campaign-style environmental governance (1–6). Recently some scholars have studied the effectiveness of CEPI (1, 3, 7–14), but most of them investigated the influence of CEPI on firm's behavior, a systematic study focusing on the pollution mitigation effects of CEPI is still limited (13). Moreover, the findings of previous studies are inconsistent due to differences in methodology and data. As such, researchers have quite differentiated views on the sustainability of CEPI. Considering that public concern about the influence of air pollution has risen to an unprecedented level in China (15), further research is needed to investigate the pollution mitigation effects of CEPI, deepen people's understanding of the effectiveness of campaign-style governance, and provide data support for the improvement of the CEPI policy.

Three roles of campaign-style governance can be identified from the existing literature. As such, it is not only necessary but also feasible to examine the pollution mitigation effects of CEPI. That is, the pollution mitigation effects can be expected considering that CEPI has the characteristics of both conventional and campaign-style governance. The first role of campaign-style governance is to show the central authorities' primary intentions and priorities to local authorities by sending clear signals (16, 17). Hence, if local authorities ignore the signals (i.e., central government's strong commitment toward environmental protection) sent by CEPI, they will be punished. For example, according to the information on the website of the Ministry of Ecology and Environment (MEE), a dyeing and weaving company in Hebei Province was convicted of polluting the environment and was fined hundreds of thousands of yuan. The person in charge was sentenced to six months in prison and was fined tens of thousands of yuan. This company replaced water samples, interfered with automatic monitoring facilities, falsified data, and evaded regulation, thus violating the *Law of the People's Republic of China on the Prevention and Control of Water Pollution*. As another example, an alloy steel limited liability company in Shandong Province illegally landfilled industrial solid waste, and therefore violated the *Law of the People's Republic of China on the Prevention and Control of Solid Waste Pollution*. The local Ecological Environment Bureau ordered it to rectify the situation, and both the company and the main person in charge were fined tens of thousands of yuan.

The second role of campaign-style governance is to go beyond conventional governance structures and improve the ability to mobilize and centralize resources on solving certain salient issues (16, 18), and CEPI is no exception. CEPI could mobilize various resources within the vertical system from top to bottom and trigger power redistribution among bureaucratic agencies, thereby stimulating the local party and government leaders to bear the “common environmental protection responsibility” and addressing the long-standing phenomenon of “formalism” in environmental governance (18, 19). The third role of campaign-style governance also is to alleviate information asymmetries (5). On the one hand, the conventional hierarchical governance structure is transformed into a flat one, thus preventing miscommunication between the central and local governments (16). On the other hand, public participation in environmental governance is promoted by conducting vigorous propaganda and providing channels for the public to express their attitudes and opinions (11).

In this regard, it is important and necessary to check the establishment of campaign-style governance in CEPI, which is conducive to deepening the reform of environmental governance system in the long run. In comparison to previous studies, the novel contributions this paper are threefold as follows. First, the study could provide more reliable results on the effectiveness of CEPI by using daily pollution data at prefecture-level city level and focusing on all batches of the first round of CEPI, which is different from previous studies utilizing provincial or monthly data or covering some rather than all batches [e.g., (3, 7, 20)]. Second, we make an empirical contribution by capturing the city-level heterogeneity (including leading industries, geographical location, and population size) and boundary conditions of policy effects. Last but not least, this study could complement the current literature on campaign-style governance by weighing in on the debate over the effectiveness of campaign-style environmental governance. The policy suggestions proposed by the study could provide a benchmark for the improvement of follow-up CEPI work and policy formulation of the central government.

**Figure 1:**
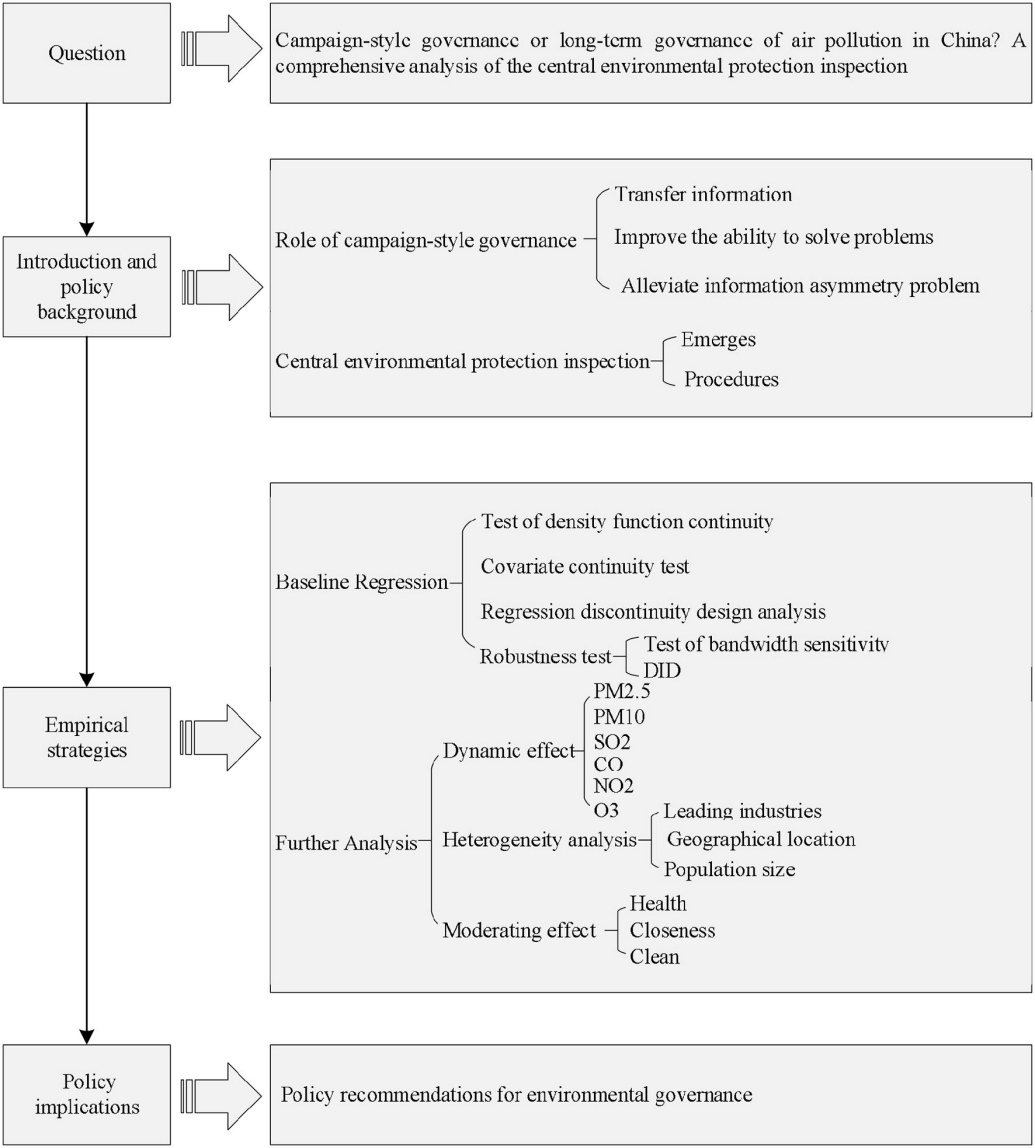
Technology steps.

The rest of this article is structured as follows. In the section 2, we outline the background of CEPI, especially how it emerges and what its procedures are. The section 3 introduces our data and methodology. The empirical results are analyzed in the section 4 and 5 presents the results of heterogeneity analysis. In the section 6, we provide the conclusion and policy implications. For a clearer understanding of the flow of the article, this paper draws the technology steps, which is shown in Figure 1.

## 2. Policy background

In China, the central government maintains its political authority over environmental policy formulation, while the local governments bear the responsibility of policy implementation (21, 22). However, for a long time, there are still many difficulties in the implementation of environmental laws and policies. One important reason for this situation is that the local government has little incentive to prioritize environmental issues. In China's cadre evaluation system, GDP growth performance is given a greater weight than other performance, which pushes local governments to prioritize economic growth even at the expense of the environment (23). To tackle the problem of weak environmental law enforcement, a number of measures have been taken by the central government over the past two decades. In December 2006, the *Decision of the State Council on Implementing Scientific Outlook on Development and Strengthening Environmental Protection* clearly stated that the pollution reduction performance should be taken as the criteria for appointing, rewarding or punishing the cadres. In 2008, the former State Environmental Protection Administration (SEPA) sets up six regional supervision centers all over the country (24). Nevertheless, the performance of such an environmental supervision system was disappointing owing to the lack of enough administrative authorization (19). In July 2015, the Central Leading Group for Comprehensively Deepening Reform (CLGCDR) approved the *Environmental Protection Inspection Plan (Trial)*, indicating the formal establishment the CEPI. In December of the same year, the first CEPI team was founded and sent to Hebei Province, making Hebei Province the first pilot region to be inspected. In July 2016, the first batch of the first round of CEPI was launched by sending eight teams to eight provinces. By September 2017, the first round of CEPI has covered all 31 provinces in mainland China (see Table 1).

**Table 1:** Dates of implementation of the first round of CEPI.

**Group**	**Inspection provinces/municipalities**	**Entry time**
Pilot	Hebei	2016.01-2016.02
1st Group	Inner Mongolia, Heilongjiang, Jiangsu, Jiangxi, Henan, Guangxi, Yunan, Ningxia	2016.07-2016.08
2nd Group	Beijing, Shanghai, Hubei, Guangdong, Chongqing, Shaanxi, Gansu	2016.11-2016.12
3rd Group	Tianjin, Shanxi, Liaoning, Anhui, Fujian, Hunan, Guizhou	2017.04-2017.05
4th Group	Jilin, Zhejiang, Shandong, Hainan, Sichuan, Tibet, Qinghai, Xinjiang	2017.08-2017.09

Source: Tan and Mao (19).

According to the *Regulations on Central Ecological Environmental Protection Inspection Work* (RCEEPIW) issued by the General Office of the CPC Central Committee and the General Office of the State Council in 2019, the CEPI teams undertake environmental protection inspection tasks on behalf of the CPC and the State Council. Moreover, the leaders of CEPI teams are consisted of currently serving or recently retired provincial and ministerial leaders, making CEPI highly authoritative (16). The focus of CEPI is to inspect how local party committees and governments implement national environmental laws and policies, address prominent environmental issues and fulfill the environmental protection obligations. In terms of the implementation process of CEPI, a typical inspection by the CEPI team lasts for 1 month. During the period, the CEPI teams conducted the inspection in various forms such as meeting with local officials, listening to reports, reading materials, communicating with local residents, receiving accusations and spot checks (3, 19). After that, the CEPI teams give feedback to the provincial party committees and governments of inspected regions and local party committees and governments are supposed to submit a rectification plan to the State Council within 30 working days. The rectification plan is subject to the supervision of the public, including rectification goals, rectification measures, rectification time, etc. (3).

It should be said that the first round of CEPI exhibited remarkable effects. According to statistics, more than 135 thousand accusation letters were received from the public, 29 thousand pollution cases were filed, and fines amounted to 1.43 billion yuan (19). Moreover, 1,527 environmental law violators were detained, 18,448 party and government officials were interviewed, and 18,199 officials held accountable (25). The second round of CEPI was launched in May 2019 and was completed in March 2022. It is too early to evaluate the effect of the second round of CEPI, but it has been several years since the end of the first round of CEPI. Therefore, it is both feasible and necessary to evaluate the effect of the first round of CEPI. In particular, the number of relevant studies is still limited, and their conclusions remain inconsistent due to different data and method. This study could provide new inspiration for the government environmental governance work.

## 3. Methodology

### 3.1. Data sources and descriptive statistics

Following prior research (26), the AQI is recognized indicators of air quality. Therefore, it is used as the dependent variable. The AQI was a comprehensive measure of air pollution and it simplifies the concentration of various air pollutants into a single index, with a higher AQI indicating worse air quality (27). The AQI was obtained from the “national air quality real-time distributing platform” of the MEE (http://106.37.208.233:20035/).

Considering that air quality is the result of the comprehensive function of many factors, especially meteorological factors, the daily meterological conditions at the city level are included in the model as control variables. More specifically, the control variables include the daily maximum and minimum temperatures (Temp_h, Temp_l), whether it is rainfall day (Rain) or snowfall day (Snow), maximum and minimum wind speeds (Wind_h, Wind_l), and legal and compensatory holidays (Holiday).

The first round of CEPI started January 2016 and ended September 2017. To minimize the impact of anthropogenic data selection on empirical results, we collected daily weather data from 285 cities in China from January 2013 to April 2019, covering a sufficient time period before and after the implementation of CEPI. The data of the starting and ending time of provincial visits by CEPI Teams are from the official website of the MEE (http://mee.gov.cn/), which is consistent with current research (18, 19). The descriptive statistical results of the variables are shown in Table 2. As shown in Table 2, the mean value of AQI is 79.5, which is at a good level. However, the minimum value of AQI is 0, while the maximum value is 1210, indicating that there are vast differences in the level of air pollution among cities in China. The control variables can be interpreted similarly.

**Table 2:** Descriptive statistics of variables.

**Variable**	***N***	**Mean**	**Std. Dev**.	**Min**	**Max**
AQI	592,900	79.50	49.22	0	1,210
Temp_h	592,899	19.41	11.04	−32	48
Temp_l	592,900	9.957	11.58	−45	37
Rain	592,900	0.343	0.475	0	1
Snow	592,900	0.0295	0.169	0	1
Wind_h	592,897	3.104	0.934	0	12
Wind_l	592,897	2.638	0.907	0	11
Holiday	592,900	0.316	0.465	0	1

### 3.2. Regression discontinuity design model

RDD was first proposed by the educational psychology literature (28), which is an observational causal identification strategy used to study the impact of a deterministic treatment assignment mechanism (29). RDD is a quasi-natural experimental estimation method because the allocation of individual intervention states near breakpoints is completely random (30). RDD has been widely applied to the assessment research of environmental policy effectiveness [see (27, 31, 32)]. Following prior studies (12, 27, 33), this study utilizes sharp regression discontinuity (SRD) model to investigate the effect of CEPI on air pollution of cities in China. According to the principle of SRD and related literature (23), the specific SRD model is set as follows:


(1) $$\begin{array}{l} Y_{ct}=\beta_{0}+\beta_{1}Inspection_{ct}+\beta_{2}f\left(x\right)+\gamma X_{ct}+\delta_{c}+\mu_{t}+\varepsilon_{ct} \end{array}$$

Among them, *Y*_*ct*_ is the dependent variable, that is, the AQI at city *c* on date *t*. *Inspection*_*ct*_ is called dummy variable of CEPI policy, indicating that city *c* was inspected on date *t*. As such, the value of *Inspection*_*ct*_ is always 1 after the inspection date *t*, and 0 otherwise. *x* is a running variable that indicates the number of days away from CEPI, *f(x)* is a polynomial function with *x* as the independent variable and it controls for unobserved trends that may affect daily air pollution concentrations. *X*_*ct*_ is a vector of control variables for city *c* on date *t*. *δ*_*c*_ denotes the city fixed effect. *μ*_*t*_ represents the time fixed effect. *ε*_*ct*_ is the error term. In particular, the main concern is *β*_1_, which captures the difference in air pollution before and after the CEPI, and can be used to analyze whether there is a “jump” in air pollution owing to the sudden shock of CEPI. If *β*_1_ is negative, indicating that CEPI indeed reduces the AQI.

## 4. Empirical results and analysis

### 4.1. Validity test of RDD

According to the basic principles of RDD, it is expected that the local air pollution index (AQI) should have a smooth trend without the presence of CEPI, but the local air pollution may have a “jump” trend under the impact of the exogenous CEPI. Some tests for validity are necessary before conducting empirical analysis. To test the applicability of the RDD, the continuity test of assignment variable and the smoothness test of covariates were conducted according to McCrary (34) and Lee and Lemieux (30).

#### 4.1.1. Continuity of assignment variable

**Figure 2:**
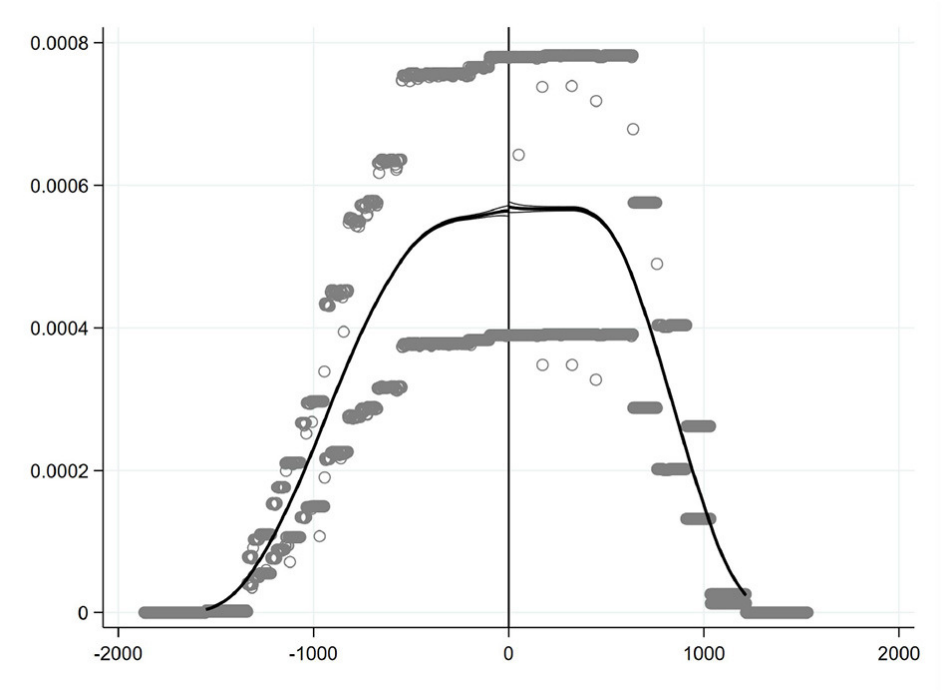
Test of density function continuity.

An important assumption of RDD is that the groups on both sides of the discontinuity are random (35). If the allocation into a treatment group or a control group can be controlled by an individual in some way, the assignment variable near the breaking point will be manipulated rather than random selected, resulting in breaking point failure. Referring to previous studies (35, 36), the density function continuity method was used to test whether the density function of assignment variable is continuous at the breaking point, and the result is illustrated in Figure 2. It is clear that the assignment variable is smooth without obvious jump at the breaking point, and the confidence interval of the estimated value of the density function on both sides of the breaking point overlaps greatly, indicating that the assignment variable is continuous at the discontinuity, that is, the entry date of the CEPI team to each city is exogenous, and the RDD method is suitable for this study. In fact, because the date of CEPI is determined by the central government, it is difficult for the local governments to manipulate this date (13).

#### 4.1.2. Covariate continuity test

Another critical premise of RDD is that covariates should not have obvious jump at the breaking point (31). If the covariates also jump at the breaking point, the change of urban air pollution cannot be simply attributed to the effect of CEPI. In this paper, the aforementioned covariates are used as the explained variables for regression analysis to judge whether the covariates are continuous at the breaking point. According to Table 3, three coefficients including Temp_l, Snow, and Holiday are not statistically significant, showing that those covariates do not jump significantly at the discontinuity. Hence, the covariate continuity assumption is basically satisfied.

**Table 3:** Covariate continuity test.

**Variables**	**Temp_h**	**Temp_l**	**Rain**	**Snow**	**Wind_h**	**Wind_l**	**Holiday**
Inspection	0.732^***^	0.253	−0.045^***^	0.001	−0.051^***^	−0.062^***^	0.004
	(4.51)	(1.20)	(−6.54)	(0.57)	(−3.92)	(−3.90)	(0.81)
Observations	592,899	592,900	592,900	592,900	592,897	592,897	592,900

z-statistics in parentheses.

*** p < 0.01.

### 4.2. Regression discontinuity design analysis

This study utilizes the RDD method to examine the effectiveness of the CEPI. According to Lee and Lemieux (30), the assignment variable shall be tried several times to select the model with the lowest Akaike Information Criterion (AIC) value during parameter estimation. Moreover, this study performed polynomial order optimization on the polynomial of *x* according to the AIC. Table 4 shows the estimation results of RDD when the 8-order polynomial is used to fit the time trend. As can be seen in Table 4, the coefficients of inspection with city and/or time fixed effects are negative, indicating that CEPI have a negative impact on the air pollution of inspected cities in China. That is, the CEPI can significantly improve air quality of cities in the inspected provinces in a short time. This finding supports the findings of previous studies [e.g., (3, 7, 23)], indicating that CEPI could still be an effective tool for policymakers to improve air quality as an campaign-style governance policy. The reason may be that CEPI has the characteristics and advantages of both conventional and campaign-style governance (18). In particular, campaign-style governance could play important roles in environmental governance (e.g., the aforementioned three roles).

**Table 4:** Baseline results based on the RDD model.

**Variable**	**AQI**		
		**(1)**	**(2)**	**(3)**
Inspection	−2.666^*^	−2.478	−2.678^*^
	(−1.65)	(−1.54)	(−1.66)
Control variables	Yes	Yes	Yes
City fixed	Yes	No	Yes
Time fixed	No	Yes	Yes
Observations	592,900	592,562	592,562

z-statistics in parentheses.

* denotes p < 0.1.

**Figure 3:**
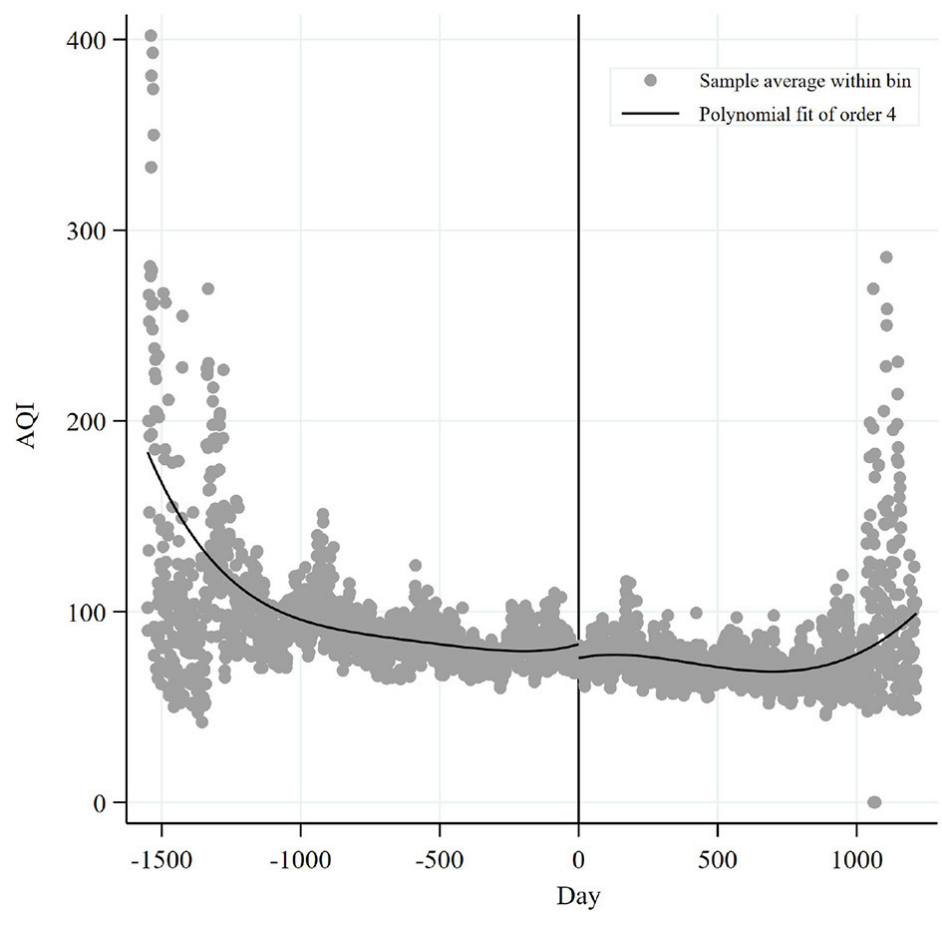
AQI fitting curve before and after the entry of the CEPI group.

As a visual demonstration, the discontinuity of the CEPI is shown in Figure 3. It is clear that the daily AQI of 285 prefecture-level cities showed a “U” shape before the CEPI, while it had a significant downward jump at the discontinuity after the CEPI, indicating that AQI had a sharp decline. Moreover, after the implementation of CEPI, AQI tends to increase, that is, CEPI is with a rebound trend in the long run (roughly 3 years). The reason for this may be that before the inspection team came and during its presence, the local governments did a good job of safeguarding air quality. Therefore, an expected effect of the inspection was achieved, and the air quality improved significantly. But after the inspection team left, the local governments slowly relaxed supervision. In such cases, enterprises are motivated to increase emissions to compensate for the loss of shutdown or production reduction during the inspection period. As a result, the air quality began to deteriorate.

### 4.3. Robustness test

#### 4.3.1. Bandwidth check

To check the robustness of research findings of this paper, several robustness tests were performed. Bandwidth is a factor affecting the robustness of the estimation results of RDD. The bandwidth robustness check was conducted according to twice, thrice and 4 times of the optimal bandwidth, respectively. The estimated results are shown in Table 5. It can be seen from Table 5 that the regression results of different bandwidths support the empirical results of RDD (Table 4). More specifically, the estimated coefficients of inspection in columns (1), (2) and (3) are significantly negative, which is consistent with the above results, indicating the robustness of above conclusions.

**Table 5:** Results of bandwidth sensitivity.

**Variables**	**Bandwidth**		
		**2 times**	**3 times**	**4 times**
Inspection	−1.138^**^	−2.740^***^	−4.080^***^
	(−2.02)	(−5.93)	(−10.19)
Observations	592,900	592,900	592,900

z-statistics in parentheses.

*** denotes p < 0.01,

** denotes p < 0.05.

#### 4.3.2. DID estimation

To control for confounding factors that may affect the daily air pollution concentrations, the DID method is employed to validate the robustness of the research finding. The DID model is set as follows:


(2) $$\begin{array}{c} Y_{ct}=\beta_{0}+\beta_{1}Inspection_{ct}+\gamma X_{ct}+\delta_{c}+\mu_{t}+\varepsilon_{ct} \end{array}$$

Where, the meanings of all symbols are consistent with those in equation (1). It is worth noting that the value of *Inspection*_*ct*_ is always 1 when the city was inspected and 0 otherwise. As such, it forms a difference-in-differences between the inspected cities and the non-inspected cities as well as before and after the inspection. The effect of CEPI on urban air pollution can be obtained by observing the coefficient of *Inspection*_*ct*_ (i.e., *β*_1_). The regression results are shown in Table 6. As shown in Table 6, the coefficients of inspection are all negative in columns (1)-(3), and they are significant in columns (2) and (3), indicating that CEPI has a negative impact on the air pollution of inspected cities in China. That is, the results of DID model are basically consistent with those of RDD.

**Table 6:** Regression results based on the DID method.

**Variables**	**AQI**		
		**(1)**	**(2)**	**(3)**
Inspection	−11.319^***^	−4.886	−3.434^*^
	(−6.478)	(−0.894)	(−2.022)
Control variables	Yes	Yes	Yes
City FE	Yes	No	Yes
Time FE	No	Yes	Yes
Observations	592,562	592,562	592,562
R-squared	0.257	0.104	0.269

Robust t-statistics in parentheses,

*** p < 0.01,

* p < 0.1.

## 5. Further analysis

### 5.1. Dynamic tests for different air pollutants

To further investigate the dynamic effect (long-term effect) of CEPI on individual pollutants including PM2.5, PM10, SO_2_, CO, NO_2_ and O_3_, we replaced the AQI with these six pollutants, with 30 days after the CEPI teams left as the first-time window and 12 dummy variables (i.e., After 1, After 2, After 3, After 4, After 5, After 6, After 7, After 8, After 9, After 10, After 11, and After 12) incorporating into the Eq. (2). The corresponding results are reported in Table 7.

**Table 7:** Dynamic effect for different air pollutants.

**Variables**	**PM_2.5_**	**PM_10_**	**SO_2_**	**CO**	**NO_2_**	**O_3_**
	**(1)**	**(2)**	**(3)**	**(4)**	**(5)**	**(6)**
After 1	−1.448	1.658^***^	0.455^**^	0.109	1.937^***^	2.368^***^
	(−0.621)	(2.891)	(2.316)	(0.221)	(15.613)	(6.206)
After 2	−1.731	−4.716^***^	−2.321^***^	−0.639	1.507^***^	5.877^***^
	(−0.738)	(−8.166)	(−11.732)	(−1.284)	(12.061)	(15.292)
After 3	−0.156	−2.775^***^	−1.494^***^	−0.731	1.375^***^	6.600^***^
	(−0.067)	(−4.841)	(−7.611)	(−1.481)	(11.088)	(17.300)
After 4	−6.592^***^	−6.706^***^	−2.779^***^	−0.138	0.922^***^	3.233^***^
	(−2.831)	(−11.699)	(−14.154)	(−0.279)	(7.436)	(8.476)
After 5	−4.398^*^	−2.836^***^	−4.544^***^	0.015	1.632^***^	−1.470^***^
	(−1.890)	(−4.952)	(−23.167)	(0.030)	(13.175)	(−3.857)
After 6	−1.229	−3.152^***^	−5.546^***^	−0.500	0.855^***^	8.015^***^
	(−0.527)	(−5.491)	(−28.206)	(−1.011)	(6.883)	(20.981)
After 7	−2.694	−7.774^***^	−5.212^***^	−0.132	0.558^***^	7.465^***^
	(−1.162)	(−13.617)	(−26.659)	(−0.269)	(4.516)	(19.651)
After 8	−7.686^***^	−13.002^***^	−4.402^***^	−0.050	0.201	5.290^***^
	(−3.316)	(−22.793)	(−22.534)	(−0.101)	(1.627)	(13.937)
After 9	−5.773^**^	−11.332^***^	−2.867^***^	−0.137	1.084^***^	1.053^***^
	(−2.489)	(−19.851)	(−14.666)	(−0.278)	(8.779)	(2.771)
After 10	−2.512	−8.339^***^	−2.987^***^	−0.091	0.606^***^	3.868^***^
	(−1.083)	(−14.614)	(−15.283)	(−0.185)	(4.908)	(10.186)
After 11	−3.858^*^	−7.146^***^	−2.488^***^	−0.485	0.444^***^	7.929^***^
	(−1.656)	(−12.464)	(−12.672)	(−0.983)	(3.579)	(20.783)
After 12	−3.116	−9.871^***^	−2.564^***^	−0.163	−0.053	0.997^***^
	(−1.344)	(−17.298)	(−13.117)	(−0.332)	(−0.429)	(2.624)
Control variables	Yes	Yes	Yes	Yes	Yes	Yes
Time fixed	Yes	Yes	Yes	Yes	Yes	Yes
City fixed	Yes	Yes	Yes	Yes	Yes	Yes
Observations	592,897	592,897	592,897	592,897	592,897	592,897
R-squared	0.005	0.131	0.163	0.000	0.284	0.350

t-statistics in parentheses.

*** p < 0.01,

** p < 0.05,

* p < 0.1.

It can be seen in Table 7 that in the first 30 days after the CEPI teams left the inspected provinces (After 1), all those six pollutants of the inspected areas did not decrease significantly. More precisely, air pollution rebounded when the CEPI teams just left for a month. It worth noting that the pollutants of PM10 and SO_2_ declined when the CEPI teams had left for 2 months (After 2) and this trend continued for 11 months. The possible reason is that when the CEPI team first leaves, the company is forced to resume production immediately due to the need for economic benefits, which leads to an increase in air pollution in the short term, however, the stringent nature of CEPI will lead to the subsequent application of greener and more efficient production methods, resulting in a sustainable impact on air. However, other four pollutants (e.g., PM2.5, CO, NO_2_ and O_3_) were not decreased or even increased when the CEPI teams had left for 2 months (After 2) or 3 months (After 3). In addition, the CO and NO_2_ did not show a downward trend from the first 30 days to the twelfth 30 days, and the O_3_ had the similar trend with CO and NO_2_ with the except of the date when the CEPI teams had left for 5 months (after 5). The PM2.5 showed an unstable trend over the twelfth months, but it indeed dropped several times.

In general, the change trend of air pollution supports the prior finding that CEPI can reduce air pollution of inspected cities, and moreover, the policy effects are sustainable. This finding is inconsistent with some studies (3, 13, 23), but consistent with some other studies (7, 16). In the meantime, this study validate an interesting finding that CEPI mainly resulted in the decrease of PM10 and SO_2_. This finding is different from Zheng and Na (23), which shows that the reduction effect of CEPI on air pollution is mainly reflected in PM2.5. However, the finding supports the conclusion that the pollution reduction of CEPI on PM2.5, CO, NO_2_ and O_3_ were not significant (19). The reason for this finding may be that PM10 and SO_2_ are the major outdoor air pollutants in China (37), and the high concentration of these two pollutants is easier to be detected (e.g., dust pollution can be easily observed as the largest pollution source of PM10; high concentration of SO_2_ produces irritating odor), so they are the focus of increasing attention. Accordingly, there will be many accusation letters concerning PM10 and SO_2_ pollution. Therefore, local governments would require polluting enterprises to strive to reduce these pollutions because CEPI teams will tract the progress of reported environmental cases. Therefore, this indeed provides a clear evidence for the establishment of campaign-style governance in CEPI. That is, CEPI could achieve short-term effects because of a series of local governments' actions intensively performed to address environmental pollution. In the meantime, CEPI could achieve long-term effects only on the issues of most concern to the public and the central government. This phenomenon is called “selective” reduction of specific pollutants by local governments in response to CEPI in some studies (e.g., 25).

### 5.2. Heterogeneity analysis

The previous analysis shows that CEPI could reduce urban air pollution significantly. However, there is reason to believe that there may be heterogeneity in leading industries, geographical location and population size considering China's vast territory and the great differences in resource endowment and economic development among cities. To capture the city-level heterogeneity of policy effects, heterogeneity analysis was conducted and the sample cities were divided into several sub-samples according to the leading industries, geographical location and population size, respectively. The estimated results are shown in Table 8. More specifically, to examine the heterogeneity of leading industries, the sample cities were divided into three sub-samples, namely, industry-oriented, service-oriented, and comprehensive cities. Similarly, the sample cities were also divided into three sub-samples, namely, eastern, central and western to capture the heterogeneity of geographical location, and small, medium and large to identify the heterogeneity of population size.

**Table 8:** Heterogeneous test.

**Variables**	**Leading industries**			**Geographical location**			**Population size**		
		**Industry**	**Service**	**Comprehensive**	**Eastern**	**Central**	**Western**	**Small**	**Medium**	**Large**
**(1)**		**(2)**	**(3)**	**(4)**	**(5)**	**(6)**	**(7)**	**(8)**	**(9)**
Inspection	−4.673^**^	0.498	−2.314	−4.022^**^	−5.002^**^	−3.254	−3.260^*^	−3.805	−4.533^**^
	(−2.747)	(0.173)	(−0.763)	(−2.559)	(−2.307)	(−1.496)	(−1.986)	(−1.638)	(−2.395)
Control variables	Yes	Yes	Yes	Yes	Yes	Yes	Yes	Yes	Yes
Time fixed	Yes	Yes	Yes	Yes	Yes	Yes	Yes	Yes	Yes
City fixed	Yes	Yes	Yes	Yes	Yes	Yes	Yes	Yes	Yes
Observations	496,448	49,278	46,836	175,251	177,197	149,385	72,254	160,124	269,455
R-squared	0.198	0.286	0.243	0.250	0.203	0.223	0.257	0.225	0.208

t-statistics in parentheses.

** p < 0.05,

* p < 0.1.

In columns (1–3), it can be seen that CEPI reduce the air pollution of the inspected industry-oriented cities, while no significant reduction can be observed in the inspected service-oriented and comprehensive cities. This result indicates that the industry-oriented cities benefited the most and CEPI mainly reduced industrial air pollution. The possible reason is that the CEPI has a more significant impact on air pollutions in industry-oriented cities, where air pollution is extremely severe due to the development of industries. In columns (4–6), we can conclude that CEPI reduce the air pollution of the inspected cities in Central and Eastern China, while no significant reduction can be observed in the inspected cities in Western China. In columns (7–9), CEPI has reduced air pollution in cities with large and small population size, but not in cities with medium population size. That is, cities in Central and Eastern China, and cities with large or small population size are more sensitive to the pollution reduction effect of CEPI, while there is still room for improvement in terms of the effectiveness of CEPI on air pollution in Western China and the medium-sized cities when compared with other cities.

### 5.3. Moderating mechanism test based on the government-business relations

Considering the dual characteristics of conventional and campaign-style governance of CEPI, government-business relations should not be ignored when investigating the boundary conditions of the effectiveness of CEPI. The government-business relations have an important impact on the effectiveness of environmental governance, especially in the context of significant institutional voids. The reason for this is that the better the government-business relations, the greater the possibility of collusion between the two (38). When local governments and enterprises collude, the government will acquiesce in the enterprises' decision to produce in a low-cost production, but this low-cost production is normally at the expense of environmental pollution and public health. When the inspectors come, the local governments shut down the polluting enterprises urgently to improve the air quality in a short-term, but when the inspectors leave, these enterprises are motivated to increase production to reduce losses. Therefore, when the local governments and enterprises collude, CEPI is not sustainable.

On the contrary, a healthy government-business relations are the key to effective environmental governance. A healthy relationship between the governments and businesses is mainly manifested in the closeness and clean of the relationship between the two. Clean government-business relations mean no collusion, rent-seeking and corruption between the governments and businesses, while close (i.e., imitate) government-business relations indicate that governments and businesses shall get along with each other in a proper way. That is, the governments will reasonably distinguish between public and private affairs and respond to the needs of enterprises with enthusiasm, thereby playing a indispensable role in regional economic development. Therefore, it is necessary to examine the impact of government-business relations on the effectiveness of CEPI.

We adopted the government-business relations indexes issued by the National Institute of Development and Strategy, Renmin University of China (http://nads.ruc.edu.cn/). The overall health index of government-business relations of Chinese cities includes two sub-indexes: the closeness (“qin”) index and the clean (“qing”) index. Each sub-index also includes several primary, secondary and tertiary indicators. We incorporated the interaction term of *Inspection* and the government-business relations indexes (including the Health, Closeness and Clean indexes) into the Eq. (2), and reported the corresponding results in Table 9. It should be noted that the average value of each index (i.e., Health, Closeness and Clean indexes) was set as the benchmark, and those greater or less than the reference value are regarded as high value group and low value group, respectively. It is clear that the six coefficients are significantly negative in columns (1)-(6), indicating that the reduction effect of CEPI on air pollution is remarkably supported, regardless of the strength (i.e., strong or weak) of government-business relations. However, it should be noted that the interaction terms of the higher values of government-business relations (i.e., Health_high, Closeness_high, and Clean_high) and CEPI have a stronger effect on reducing air pollution, implying that healthy, close and clean government-business relations are indeed conducive to reducing air pollution. Hence, to win the blue-sky war in the long-term, it is quite important and necessary to optimize government-business relations in to the process of conducting CEPI.

**Table 9:** Regression results of the moderating mechanism test based on the government-business relations.

**Variables**	**AQI**					
		**(1)**	**(2)**	**(3)**	**(4)**	**(5)**	**(6)**
Inspection × Health_high	−0.680^***^					
	(−3.387)					
Inspection × Health_low		−0.205^***^				
		(−3.722)				
Inspection × Closeness_high			−1.295^***^			
			(−8.722)			
Inspection × Closeness_low				−0.280^***^		
				(−3.724)		
Inspection × Clean_high					−0.230^***^	
					(−4.730)	
Inspection × Clean_low						−0.120^***^
						(−3.755)
Control variables	Yes	Yes	Yes	Yes	Yes	Yes
Time fixed	Yes	Yes	Yes	Yes	Yes	Yes
City fixed	Yes	Yes	Yes	Yes	Yes	Yes
Observations	133,608	151,504	128,440	156,672	142,056	143,056
R-squared	0.187	0.196	0.204	0.180	0.165	0.225

t-statistics in parentheses.

*** p < 0.01.

## 6. Conclusions and policy implications

In this study, we examine the effect of CEPI on urban air pollution by using the daily air pollution data of 285 cities in China from January 2013 to April 2019, and taking the CEPI as a quasi-natural experiment. The main findings of the paper are summarized as follows: First, the baseline SRD estimation result shows that the first round of CEPI reduced the air pollution (AQI index) of cities in the inspected provinces in a short time. Several robustness checks support this conclusion. Second, there was no retaliatory rebound in the concentration of the pollutants about which the public is most concerned (i.e., PM10 and SO_2_) due to the departure of the CEPI teams. That is, the sustainability of CEPI has been verified in reducing these two pollutants, but not in AQI and other pollutants (i.e., PM2.5, CO, NO_2_ and O_3_). Third, there is vast heterogeneity in the effectiveness of CEPI. More specifically, CEPI is only effective in reducing air pollutants of industry-oriented cities, cities in Central and Eastern China, and cities with large or small population size. Last but not least, the moderating mechanism test indicates that healthy, close and clean government-business relations could strength the pollution mitigation effect of CEPI.

In general, CEPI is effective in reducing urban air pollution in the short-term, but this pollution mitigation effect is not significant in the long run. This may be due to the fact that the local governments are inclined to adopt a “one-size-fits-all” approach to achieve the goal of reducing pollution by shutting down polluting enterprises during the inspection. But after the inspection teams leave, the local governments, which are aimed at economic growth, normally acquiesce in enterprises' environmental pollution behavior. Therefore, it is necessary to establish a long-term mechanism for CEPI. An interesting finding is that the emission reduction effect of CEPI on various pollutants is heterogeneous, and CEPI could achieve long-term effects only on the issues of most concern to the public (i.e., PM10 and SO_2_ pollution). Moreover, the effectiveness of CEPI also shows city-level heterogeneity according to leading industries, geographical location and population size. This implies that regional endowment factors indeed affect the effectiveness of CEPI.

Several policy implications can be drawn from the empirical results. First, considering the initial success of the CEPI, it is necessary to continue to implement CEPI for a long time to come. More specifically, the central government should promote the normalization of the CEPI by increasing the randomness of inspection in terms of inspection time and areas. In the meantime, the existing environmental governance measure (e.g., environmental protection inspection and environmental protection interview) should be comprehensively applied to achieve effective connection and supplement to overcome the shortcoming of campaign-style governance. Moreover, an environmental monitoring system based on advanced software and hardware facilities such as satellite remote sensing, infrared identification and cloud computing urgently needs to be established to monitor the information of various air pollutants in real time, thereby providing a reference for the “looking back” of CEPI. Second, to enhance the sustainability of CEPI in reducing air pollution, it is necessary to develop a new environmental governance mechanism that combines the advantages of campaign-based governance and conventional governance. This is particularly important when considering the costs of campaign-style governance. Third, the central government should adopt different CEPI measures according to regional endowment factors (i.e., precision inspection), instead of adopting a “one-size-fits-all” approach. For instance, the unified inspection time span of each province can be adjusted according to specific conditions. Finally, citizens' awareness of environmental protection should be strengthened by setting more public complaint channels. In the meantime, the pollutant information of high-polluting industries and enterprises shall be disclosed to the public in time. As such, a bottom-up supervision mechanism can be established to form effective supervision and restriction of the behaviors of polluting enterprises and local governments. Therefore, the possible of collusion between local governments and enterprises can be minimized.

This study is not exempt from limitations. The first limitation is that this study investigated the relationship between CEPI and urban air pollution, but it did not explore the micro mechanism of how CEPI influences urban air pollution due to the obstacle of data. Another limitation is that this study took the several batches of the first round of CEPI as a whole, thereby ignoring the possible differences of different batches of inspection. Future research is thus needed to explore the influencing mechanisms and give fine-grained quantitative analysis results concerning the effects of different batches of CEPI.

## Data availability statement

The original contributions presented in the study are included in the article/supplementary material, further inquiries can be directed to the corresponding author.

## Author contributions

YF: conceptualization, methodology, and formal analysis. CC: data curation and writing—original draft, visualization, and investigation. SH: writing—review and editing, supervision, and resources. AC: software, data, and variables. All authors contributed to the article and approved the submitted version.
